# Molecular identification and characterization of rhodaneses from the insect herbivore *Pieris rapae*

**DOI:** 10.1038/s41598-018-29148-5

**Published:** 2018-07-17

**Authors:** Anna-Maria Steiner, Christine Busching, Heiko Vogel, Ute Wittstock

**Affiliations:** 1 Institute of Pharmaceutical Biology, Technische Universität Braunschweig, Mendelssohnstr. 1, 38106 Braunschweig, Germany; 2 Department of Entomology, Max Planck Institute for Chemical Ecology, Hans-Knöll-Str. 8, 07745 Jena, Germany

## Abstract

The association of cabbage white butterflies (Pieris *spec*., Lepidoptera: Pieridae) with their glucosinolate-containing host plants represents a well-investigated example of the sequential evolution of plant defenses and insect herbivore counteradaptations. The defensive potential of glucosinolates, a group of amino acid-derived thioglucosides present in plants of the Brassicales order, arises mainly from their rapid breakdown upon tissue disruption resulting in formation of toxic isothiocyanates. Larvae of *P*. *rapae* are able to feed exclusively on glucosinolate-containing plants due to expression of a nitrile-specifier protein in their gut which redirects glucosinolate breakdown to the formation of nitriles. The release of equimolar amounts of cyanide upon further metabolism of the benzylglucosinolate-derived nitrile suggests that the larvae are also equipped with efficient means of cyanide detoxification such as β-cyanoalanine synthases or rhodaneses. While insect β-cyanoalanine synthases have recently been identified at the molecular level, no sequence information was available of characterized insect rhodaneses. Here, we identify and characterize two single-domain rhodaneses from *P*. *rapae*, PrTST1 and PrTST2. The enzymes differ in their kinetic properties, predicted subcellular localization and expression in *P*. *rapae* indicating different physiological roles. Phylogenetic analysis together with putative lepidopteran rhodanese sequences indicates an expansion of the rhodanese family in Pieridae.

## Introduction

Plants have evolved various defense mechanisms against herbivore attacks. In turn, insects have developed strategies to overcome these defenses. The repeated sequence of reciprocal defensive and counterdefensive adaptations in plants and herbivores is also known as “evolutionary arms race” and represents a central idea of the theory of co-evolution proposed by Ehrlich and Raven in 1964^1^. A very well investigated example of a plant-herbivore relationship that shows such reciprocal responses is the interaction of cabbage white butterflies (Pieris *spec*., Lepidoptera: Pieridae) with their host plants of the order Brassicales^2,3^. As a common feature, these plants are all defended by a group of small molecules, i.e. specialized metabolites known as glucosinolates^4^. Glucosinolates are largely non-toxic thioglucosides with aliphatic or aromatic side chains which are broken down by co-occurring thioglucosidases, the myrosinases, when plant tissue is damaged^5^. This gives rise to the formation of isothiocyanates which are toxic to many organisms and provide protection against a wide range of plant enemies^6,7^. Pieris *spec*. such as the small white, *Pieris rapae*, have acquired biochemical and sensorial adaptations that enable them to exploit glucosinolate-containing plants as their sole host plants for oviposition and larval feeding, i.e. they have become specialized on these plants despite their powerful chemical defense^8–11^. An evolutionary key innovation that allowed the corresponding Pierid subfamily, the Pierinae, to occupy glucosinolate-containing plants has been identified as the nitrile-specifier protein (NSP) that is expressed in the larval gut and redirects glucosinolate breakdown to formation of nitriles^12,13^. The host shift of Pierinae from Fabales to Brassicales was followed by expansion and species diversification within the Pierinae^13^. This happened only shortly after the evolution of the glucosinolate-myrosinase system as the characteristic chemical defense of the Brassicales order about 80 million years ago^13,14^.

Previous research has established that the fate of nitriles produced by larval NSP depends on the structure of the side chain. While aliphatic nitriles are excreted without further processing in larvae of *P*. *rapae*, nitriles derived from benzylic glucosinolates are further metabolized^12,15–18^. In the case of phenylacetonitrile derived from benzylglucosinolate, the major route of metabolism in *P*. *rapae* starts with NADPH-dependent α-hydroxylation by microsomal gut enzymes^18^. The resulting α-hydroxynitrile is unstable and decomposes to benzaldehyde and cyanide^18^, a universal and highly potent respiration toxin^19^. Consequently, feeding on a benzylglucosinolate-containing plant such as *Tropaeolum majus* (Tropaeolaceae; 5 µmol/g benzylglucosinolate) exposes larvae to toxic amounts of cyanide, yet without known ill effects^18^. Aromatic glucosinolates such as benzylglucosinolate are widespread in basal Brassicales^20^. Therefore, an efficient cyanide detoxification mechanism of the larvae might have been a prerequisite for the host shift^18^. Activities of cyanide detoxification enzymes have been detected in a range of arthropods^21–25^. These include β-cyanoalanine synthases, catalyzing the substitution of the sulfhydryl group of cysteine by cyanide, and rhodaneses, catalyzing the transfer of sulfur to cyanide. The presence of isotopically labelled β-cyanoalanine and thiocyanate (SCN^−^) in *P*. *rapae* larvae after fumigation with [^15^N]HCN indicated that larvae detoxify cyanide by both β-cyanoalanine synthases and rhodaneses^18^. Both enzyme activities were detected in larval gut tissue^26,27^. While three β-cyanoalanine synthases have recently been identified in *P*. *rapae*^26^, no rhodanese sequences from invertebrates were available until recently when the single-domain rhodaneses MnRDH1 and MnRDH2 were cloned and characterized from the oriental river prawn (*Macrobrachium nipponense*, Crustacea/Malacostraca)^28,29^.

Rhodaneses (thiosulfate:cyanide sulfurtransferases; EC 2.8.1.1; thiosulfate sulfurtransferases) catalyze the transfer of sulfur from thiosulfate to cyanide leading to the formation of thiocyanate and sulfite. A major role of rhodaneses in detoxification of cyanide is supported by the mitochondrial localization of many rhodaneses^30,31^ and by the high concentration of bovine rhodanese in cyanide-exposed tissues, like hepatocytes, kidney cells or lung epithelial cells, as shown by immunohistochemical localization^32^. However, the ubiquitous distribution of rhodanese activity in organisms that are normally not exposed to high levels of cyanide and the low physiological concentration of the substrate thiosulfate make the case for alternative physiological functions^23,33^. These include a role in sulfur and selenium metabolism, the synthesis of iron-sulfur proteins, and redox regulation^33,34^). Mammalian rhodaneses such as the well-investigated bovine liver rhodanese are composed of two domains of similar length but with low amino acid sequence similarity. Only the C-terminal domain is catalytically active^35^. The two domains are thought to have evolved through duplication of an ancestral rhodanese gene^35^. This hypothesis is supported by the identification of single domain rhodaneses from bacteria, such as GlpE from *Escherichia coli*^36^. While the three-dimensional structure of rhodaneses is highly conserved, the amino acid sequence identity among rhodaneses from different organisms is rather low, which makes the identification of new rhodanese sequences challenging^33,37^.

Based on the observation that larvae of *P*. *rapae* perform well on plants which either produce cyanide upon damage or give rise to cyanide formation inside the larvae^18^, this study was aimed at identifying cyanide detoxification enzymes, namely rhodaneses, from *P*. *rapae*. As efficient cyanide detoxification mechanisms might have likely been a prerequisite for the host plant shift from Fabales to Brassicales, we were especially interested in the catalytic properties of rhodaneses in comparison to β-cyanoalanine synthases from *P*. *rapae* as well as rhodanese diversification within Lepidoptera.

## Results

### Transcriptome sequencing identified candidate P. rapae rhodanese cDNAs

Two entries annotated as thiosulfate sulfurtransferase were retrieved from a *P*. *rapae* RNAseq transcriptome database and designated as *P*. *rapae* thiosulfate sulfurtransferase 1 (*PrTST1*) and *P*. *rapae* thiosulfate sulfurtransferase 2 (*PrTST2*) (see Supplementary Fig. S1). The entries consisted of partial coding sequence and, in case of PrTST1, 5′-untranslated region (UTR). Complete coding sequences were identified by 3′- and 5′-RACE and entire open reading frames (ORF) confirmed in one PCR using larval gut cDNA as a template (see Supplementary Fig. S1). The putative rhodanese cDNAs encoded polypeptides of 171 (PrTST1) and 126 (PrTST2) amino acids with a molecular weight of 19.4 kDa (PrTST1) and 14.2 kDa (PrTST2). The amino acid sequence identity between PrTST1 and PrTST2 was 47%, whereas the identity to rhodanese sequences from other organisms was below 35% (Table 1). Both sequences comprise a conserved cysteine that is predicted to be part of the active site (PrTST1: Cys-135, PrTST2: Cys-90; NCBI Conserved Domain Database). Based on analysis with TargetP^38^ and MitoProt^39^, only PrTST1 possesses an N-terminal mitochondrial target peptide of 51 amino acids.

**Table 1:** Amino acid sequence identity of PrTST1 and PrTST2 to rhodaneses and putative rhodaneses from other organisms.

Organism/protein (accession no.)	Identity to PrTST1 (%)	Identity to PrTST2 (%)
*B*. *taurus* rhodanese (AAA30753.1)	15.8	20.3
*C*. *elegans* CAB02870.1	15.0	12.1
*M*. *nipponense* MnRDH1 (ALJ10568.1)	29.4	33.6
*M*. *nipponense* MnRDH2 (ATD13076.1)	25.7	29.8
*A*. *thaliana* STR18 (BAB10422.1)	27.6	25.7
*E*. *coli* glpE (AAA23889.1)	19.7	22.7

### The cloned cDNAs encode rhodaneses with distinct kinetic properties

**Figure 1:**
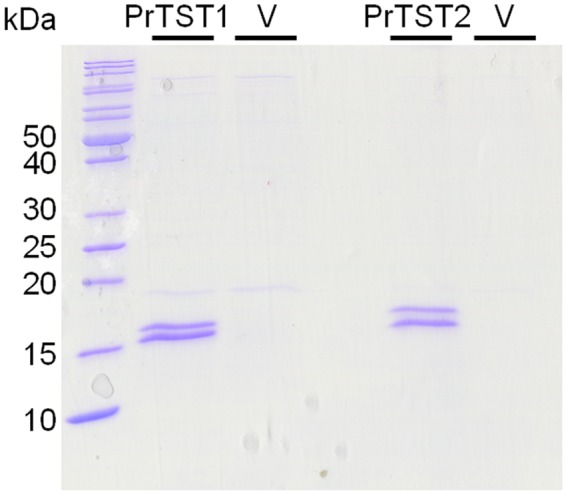
Heterologous expression and purification of PrTST1 and PrTST2. Proteins were expressed in *E. coli* in fusion with an N-terminal Strep-tag (PrTST1 without its target peptide). As a control, *E. coli* transformed with the expression vector without insert were treated the same. Coomassie Brilliant Blue R250-stained SDS-PAGE of 2 µg of purified recombinant enzymes compared with the same volumes of vector control (V). The full-length gel image is shown in Supplementary Fig. S3.

To test if the putative rhodanese cDNAs encode proteins with rhodanese activity, we expressed them heterologously in *E*. *coli* in frame with the sequence for an N-terminal Strep tag. As expression of the entire ORF of *PrTST1* scarcely yielded any recombinant protein (also when expressed with C-terminal Strep tag), PrTST1 was expressed without its mitochondrial target peptide, i.e. as a protein of 121 amino acids corresponding to 13.7 kDa plus N-terminal Strep tag. Successful protein expression and purification was confirmed by SDS-PAGE. Protein bands of about 15 kDa were evident in samples from *E*. *coli* transformed with either of the expression constructs, but not in control samples from *E*. *coli* transformed with the expression vector without insert (Fig. 1). Thus, they represented the recombinant proteins PrTST1 and PrTST2. The bands often appeared as double bands (Fig. 1) likely due to re-oxidation of a proportion of the protein.

**Figure 2:**
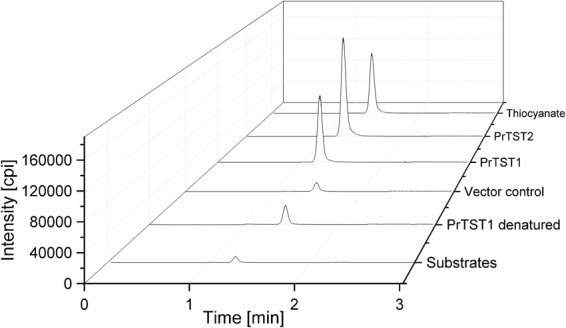
Thiocyanate formation by rhodaneses from P. rapae. Purified PrTST1, PrTST2, heat denatured PrTST1, or an equal volume of empty vector control were incubated with cyanide and thiosulfate for 10 min. In addition, cyanide and thiosulfate were incubated under identical conditions without added protein. The reaction mixtures and a thiocyanate standard were analyzed by HPLC-MS. The HPLC-MS trace for m/z 57.9 is depicted.

**Figure 3:**
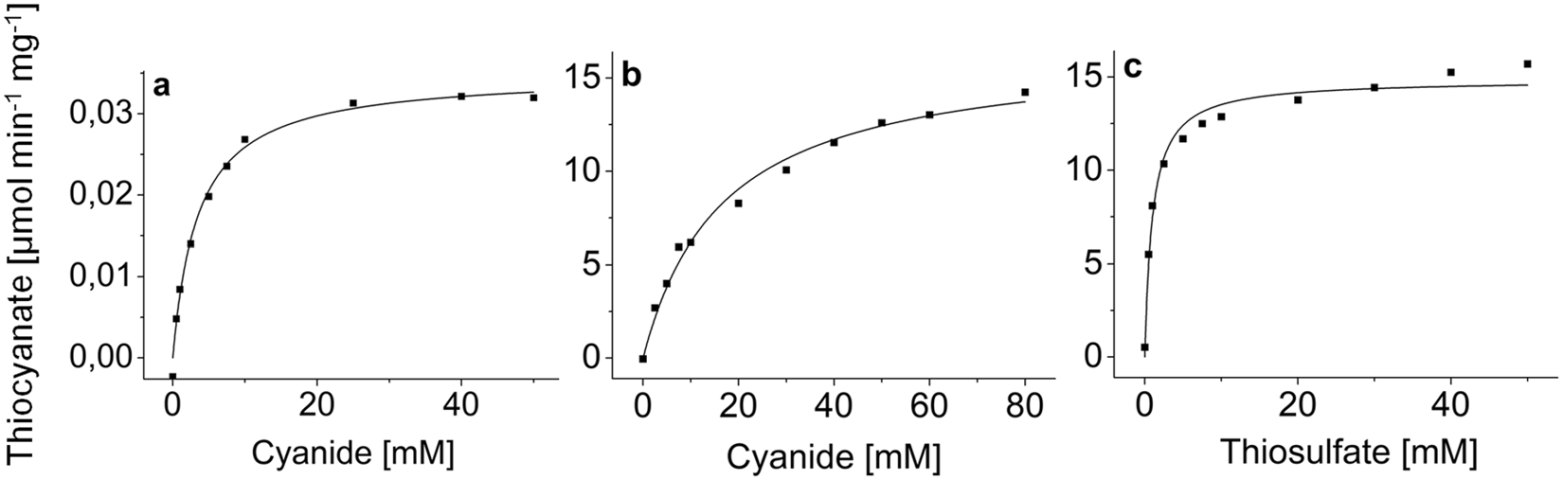
Kinetics of PrTST1 and PrTST2. (**a**) PrTST1 with cyanide as a substrate, (**b**) PrTST2 with cyanide as a substrate and (**c**) PrTST2 with thiosulfate as a substrate. The purified recombinant enzymes were incubated with 50 mM thiosulfate or cyanide while the concentration of the other substrate was varied. Thiocyanate formation was determined colorimetrically and the mean value of three technical replicates is depicted for one out of three independent experiments. The curves were generated by nonlinear fitting to the Michaelis-Menten equation.

After incubation of purified proteins with KCN and sodium thiosulfate, reaction mixtures were analyzed for thiocyanate by HPLC-MS. In reactions carried out with PrTST1 and PrTST2, the thiocyanate ion (*m/z* 57.9) was detected with high intensity, i.e. far above the background obtained when the substrates were incubated without any protein (Fig. 2). When equal amounts of heat-denatured enzyme or equal volumes of elution fractions from extracts of *E*. *coli* transformed with empty vector control were used instead of PrTST1 and PrTST2, only background levels of thiocyanate were formed (Fig. 2). Rhodanese activity of PrTST1 and PrTST2 was further confirmed through colorimetric detection of thiocyanate after incubation of PrTST1 or PrTST2 with KCN and sodium thiosulfate (Fig. 3).

To determine enzyme kinetics, PrTST1 and PrTST2 were subjected to a rhodanese assay with varying substrate concentrations and the formation of thiocyanate was determined colorimetrically. For PrTST1 and cyanide as a substrate, substrate dependency of activity followed Michaelis-Menten-kinetics with a *K*_m_ of 3.35 mM (Fig. 3a, Table 2). For thiosulfate as a substrate, substrate saturation could not be obtained. The reaction velocity of PrTST2 depended on cyanide and thiosulfate concentrations according to the Michaelis-Menten equation (Fig. 3b,c). The *K*_m_ value for cyanide was almost 20-fold higher than that of PrTST1, but PrTST2 seemed to be much more efficient than PrTST1 (Table 2).

**Table 2:** Kinetic constants of PrTST1 and PrTST2.

	KCN		Thiosulfate	
	V_max_ [µmol min^−1^ mg^−1^]	*K*_m_ [mmol l^−1^]	V_max_ [µmol min^−1^ mg^−1^]	*K*_m_ [mmol l^−1^]
PrTST1	0.03 ± 0.00	3.35 ± 0.50	n. d.	n. d.
PrTST2	25.23 ± 2.60	56.16 ± 10.30	14.17 ± 1.45	0.84 ± 0.27

After incubation of purified recombinant PrTST1 and PrTST2 with KCN and thiosulfate for 10 min, thiocyanate formation was determined. KCN was used at 50 mM when thiosulfate concentrations were varied, and thiosulfate was used at 50 mM when KCN concentrations were varied. Means ± SEM are given as determined in n = 3 independent expression experiments. n.d.: not detectable.

### PrTST1 and PrTST2 are differentially expressed

**Figure 4:**
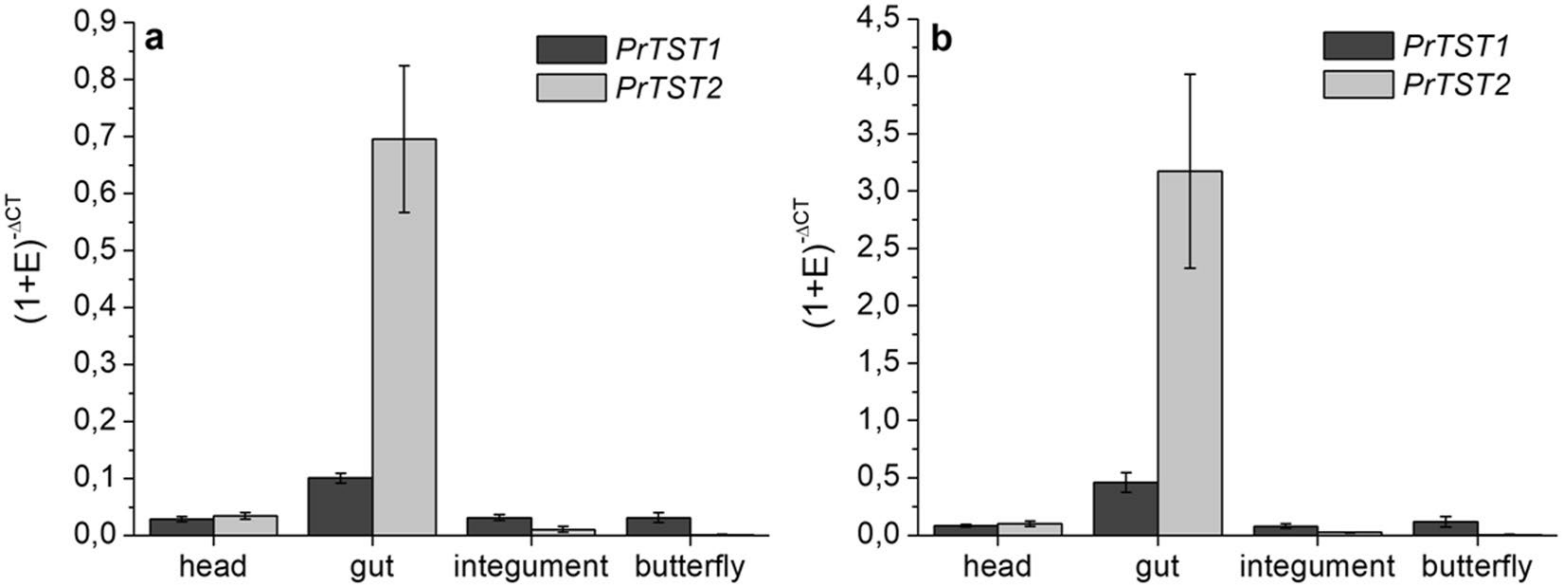
Expression of *PrTST1* and *PrTST2* in *P*. *rapae*. Equal amounts of RNA from different larval parts and imagos were used for cDNA synthesis. qPCR was carried out using SYBR Green for quantification. The CT values of *PrTST1* and *PrTST2* amplifications were normalized to *EF1α* (**a**) or *GAPDH* (**b**) as reference (Ref) gene. The expression level of the gene of interest (GOI) in each tissue was expressed as (1 + E)^−ΔCT^ = (1 + E_Ref_)^CTRef^/(1 + E_GOI_)^CTGOI^ (E, primer efficiency). Means ± SD of n = 3 biological replicates.

To investigate in which tissues and stages the two identified rhodanese genes are expressed, we analyzed transcript levels of *PrTST1* and *PrTST2* in gut tissue, integument and head of late-instar larvae and in adult butterflies by using qPCR. This showed that both genes were expressed mainly in the gut tissue of the larvae with much higher transcript levels of *PrTST2* as compared to *PrTST1* (Fig. 4). Both transcripts were also detected in head and integument, but only at low levels (Fig. 4). Imagines were found to express *PrTST1*, but not *PrTST2* (Fig. 4).

### Phylogenetic analysis indicates lineage-specific expansion of the rhodanese gene family in Pieridae

**Figure 5:**
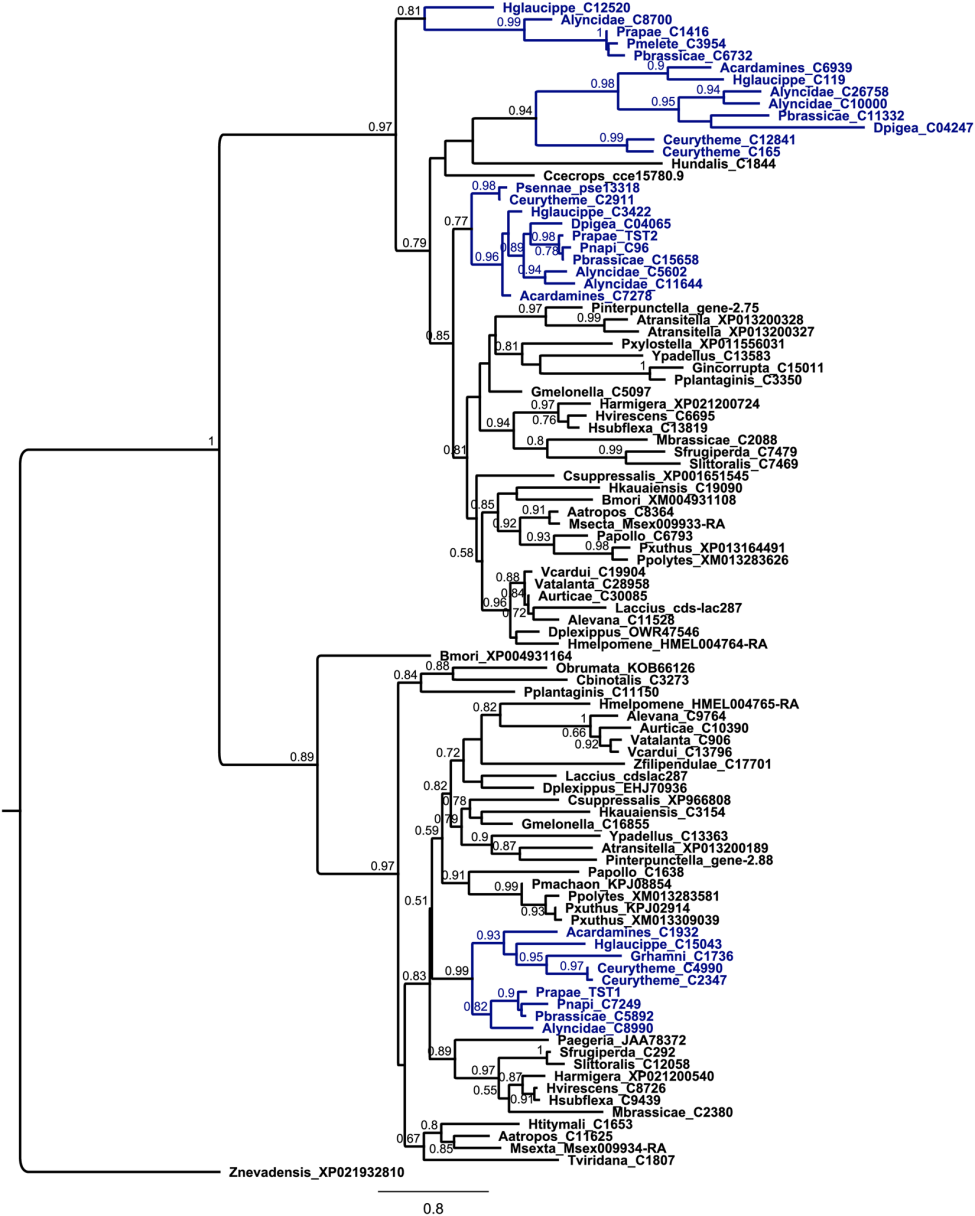
Phylogenetic relationships of rhodaneses and putative rhodaneses from Lepidoptera. To minimize the size of the tree, only rhodanese sequences from Lepidoptera were included in this figure. A maximum likelihood analysis was performed with 1,000 bootstrap replicates with bootstrap values indicated next to the branches. Bootstrap values below 50% are not shown. The tree is represented as a cladogram with branch lengths and rooted with the *Zootermopsis nevadensis* (Isoptera) rhodanese sequence (GenBank Accession Number XP021932810) as outgroup. Rhodanese sequences from Pieridae species are shown in blue. Details of the species names and sequences used for the analysis as well as accession numbers are provided in Supplementary Table S1.

To identify rhodanese proteins from other Lepidopteran species, *P*. *rapae* PrTST1 and PrTST2 amino acid sequences were used as query for BLAST searches in Genbank, Lepidopteran Genome databases (Lepbase) and in-house transcriptome assemblies. We initially attempted to combine all available Lepidopteran rhodanese protein sequences with other insect sequences as well as plant and bacterial homologs to perform a global Maximum Likelihood-inferred phylogenetic analysis. However, since the bacterial, plant and insect-derived sequences each formed distinct clusters with good node support (see Supplementary Fig. S2), we subsequently focused on the Lepidopteran rhodanese candidates only. Protein sequences of species from 17 lepidopteran families were aligned using MUSCLE and phylogenetic trees were generated based on the manually curated protein sequence alignments in Geneious using a Maximum Likelihood method and Bayesian inference. In general, the Maximum Likelihood and Bayesian analysis gave similar tree topologies with nodes being well supported in both methods. Phylogenetic analysis revealed two large subclades each of which contained one of the characterized rhodaneses from *P*. *rapae*. PrTST1 formed an orthologous cluster with other lepidopteran rhodanese-like proteins. This corresponded well with the phylogenetic relationships among the species and did not indicate any obvious species-specific gene duplications (Fig. 5). Although the majority of lepidopteran sequences in the clade with PrTST2 also showed true orthology and reflected species phylogenetic relationships, this was not true for the Pieridae-derived sequences in this clade. Within this clade, several paralogs were present in several Pieridae species (e.g. in *Pieris* spp. and *Colias eurytheme*) indicating lineage-specific expansions in the Pieridae rhodanese gene family.

## Discussion

Due to the frequent occurrence of cyanogenic compounds such as cyanogenic glycosides in plants, insect herbivores have long been thought to be equipped with efficient means of cyanide detoxification^40,41^. Although rhodanese and β-cyanoalanine synthase activities have been detected in herbivores of various invertebrate taxa^25^, the first β-cyanoalanine synthases from animals have been identified only recently^26,42,43^. This allowed studies on their evolutionary history which revealed their recruitment from bacteria by horizontal gene transfer in case of the enzymes from the two-spotted spider mite, *Tetranychus urticae* (Trombidiformes: Tetranychidae)^43^. Lepidopteran β-cyanoalanine synthases likely arose through the same or a later horizontal gene transfer event as they cluster together with the mite enzymes within bacterial sequences and separately from other animal and plant sequences in phylogenetic analyses^26,27^. In case of rhodaneses, the first enzymes from invertebrates, MnRDH1 and MnRDH2 from the oriental river prawn, have been identified recently^28,29^. Here, we identify the first rhodaneses from insects and provide evidence for their activity as thiosulfate:cyanide sulfurtransferases by enzyme characterization after heterologous expression and purification. In contrast to the phylogeny of β-cyanoalanine synthases, that of rhodaneses reflects general organismic phylogeny with no indication for horizontal gene transfer events.

Previous work demonstrated expansion and diversification of β-cyanoalanine synthases in the Pierid family of lepidopteran insects^27^. While β-cyanoalanine synthases of the BSAS2 group were present in diverse lepidopteran species, enzymes of the BSAS3 and BSAS1 groups appeared to be specific for Pierids. Interestingly, the phylogenetic analysis of rhodaneses in Lepidoptera shows that Pieridae also seem to possess additional rhodanese homologs. The two rhodaneses PrTST1 and PrTST2 from *P*. *rapae*, which were identified in this work, belong to two different clades in the phylogenetic tree. Both clades roughly mirror species phylogenies and contain one ortholog from each investigated species. However, the clade with PrTST2 comprises up to two additional homologs from several of the investigated Pierid species. This includes an additional *P*. *rapae* rhodanese candidate (Prapae_C1416, Fig. 5). Although we cannot exclude that the absence of paralogs from other families in the PrTST2 clade is due to the limited availability of sequence data, this result indicates that species or lineage-specific gene duplications followed by sub/neofunctionalization could have led to diversification of rhodaneses within the PrTST2 clade in the Pieridae. It maybe surprising that a species specialized on cyanogenic glucoside-containing plants, *Zygaena filipendulae* (Lepidotpera: Zygenidae), appears to possess only a single rhodanese (and one β-cyanoalanine synthase)^27^ based on the present knowledge. As a possible explanation, this species relies mostly on an ‘avoidance strategy’, i.e. a combination of behavioural, physiological and biochemical mechanisms that allows the larvae to prevent cyanide release during feeding^44^. Thus, the larvae encounter only low amounts of cyanide despite their cyanogenic hosts, in contrast to *P*. *rapae* larvae in which cyanide is formed in gut tissue upon nitrile metabolism. However, based on the different kinetic properties and predicted subcellular localization of PrTST1 and PrTST2, rhodaneses of the two clades may have different physiological functions. More enzymes need to be characterized to find out if orthologs within each of the clades share biochemical properties and if diversification within the PrTST2 clade resulted in new specificities, reactivities or changed kinetic properties. Moreover, it will be interesting to clarify if all sequences of putative insect rhodaneses included in our study represent single domain rhodaneses (such as PrTST1 and PrTST2) or if diversification within Lepidoptera or certain families and lineages has led to the appearance of rhodaneses with two domains as commonly found in mammals.

In agreement with a role in cyanide detoxification, PrTST1 is expressed mainly in gut tissue, is predicted to be localized in the mitochondria and possesses a 17-fold higher affinity for cyanide than PrTST2. However, when compared to the β-cyanoalanine synthases PrBSAS1-PrBSAS3 from *P*. *rapae* and β-cyanoalanine synthases from other lepidopteran species^26,27^, its *K*_m_ is relatively high (>3 mM vs. 0.02–8 mM for β-cyanoalanine synthases) and the V_max_ low (0.03 µmol min^−1^ mg^−1^ vs. 0.05–17 µmol min^−1^ mg^−1^ for β-cyanoalanine synthases). This puts a possible role in cyanide detoxification into question. Only PrTST1, but not PrTST2, is expressed in the imago, albeit at low levels. A more detailed analysis of different organs of the imago is required to get hints as to whether PrTST1 could enable the butterflies to consume nectar of cyanogenic plants with impunity by converting released cyanide to thiocyanate. In case of PrTST2, which is presumably a cytosolic enzyme, the very low affinity for cyanide also argues against a role as a classical rhodanese despite its high V_max_ value and its predominant and high expression in gut tissue. For some rhodanese-like proteins, a higher affinity for mercaptopyruvate than for thiosulfate has been demonstrated, identifying them as mercaptopyruvate-sulfur-transferases (MST). These proteins share high sequence identities with rhodaneses and can also transfer sulfur to cyanide^45^. In contrast to rhodaneses, which are primarily located in mitochondria, MSTs are mostly cytosolic enzymes^45^. Besides other functions, MSTs are also thought to act in concert with rhodaneses in the detoxification of cyanide. In addition to the direct detoxification of cyanide in the cytosol, MSTs are able to produce sulfane sulfur which could possibly be used as a substrate by rhodaneses^46^. Whether PrTST1 and PrTST2 can convert mercaptopyruvate or other substrates and if this conversion is more efficient than that of thiosulfate and cyanide remains subject of future investigations.

Besides a function in cyanide detoxification, single-domain rhodaneses have been associated with responses to stress conditions^28,29,47^. Other postulated functions include a role in sulfur oxidation and metabolism and formation of iron-sulfur clusters^48,49^. The identification of single domain rhodaneses from insects, their distinct kinetics and expression profiles as well as the expansion of the rhodanese family in the Pieridae raise questions about general physiological functions of rhodaneses in insects and about more specific roles that they may have acquired in Pierid species in response to environmental challenges or in the course of host plant adaptation.

This study was initiated based on the observation that larvae of *P*. *rapae* encounter high levels of cyanide when they consume a benzylglucosinolate-containing plant, the demonstrated conversion of isotopically labelled cyanide to thiocyanate by larvae *in vivo* and the presence of rhodanese activity in larval extracts *in vitro*^18,27^. We demonstrate here that *P*. *rapae* larvae express two rhodaneses in their gut, PrTST1 and PrTST2, which both are able to catalyze the transfer of sulfur from thiosulfate to cyanide and could therefore be involved in detoxification of cyanide generated in the gut upon benzylglucosinolate breakdown and further metabolism. Future research will have to investigate if PrTST1 and PrTST2 as well as other insect rhodaneses play multiple roles in diverse processes and how such functions are coordinated to ensure high performance. Furthermore, the expansion of the rhodanese family in Pieridae deserves attention, not least because of the interesting parallel to the diversification in Pieridae of the β-cyanoalanine synthases, the other group of cyanide detoxification enzymes which have long been discussed to be involved in shaping plant-insect interactions.

## Methods

### Insects

A *P*. *rapae* colony was maintained on brussels sprout plants (*Brassica oleracea* var. *gemmifera*) as described previously^26^. Other species were kept on an appropriate host plant or artificial diet as indicated in Supplementary Table S1, i.e. only glucosinolate-specialists were grown on glucosinolate-containing plants.

### Transcriptome sequencing

Transcriptomes of *P*. *rapae* and further moth and butterfly species (Supplementary Table S1) were obtained by isolating RNA from actively feeding larvae, and performing RNAseq using Illumina sequencing. Transcriptome sequencing was carried out using poly(A)+ enriched RNA fragmented to an average of 150–200 nucleotides. Sequencing was done by the Max Planck Genome Center Cologne (MPGCC) or GATC Biotech (www.gatcbiotech.com) on Illumina HiSeq2500 Genome Analyzer platforms using paired-end (2 × 100 or 2 × 125 bp) reads. Quality control measures, including the filtering of high-quality reads based on fastq file scores, the removal of reads containing primer/adapter sequences, and trimming of the read length, as well as subsequent de novo transcriptome assemblies were carried out using CLC Genomics Workbench software (http://www.clcbio.com), selecting the presumed optimal consensus transcriptome as previously described^50^. The transcriptomes were annotated using BLAST, Gene Ontology and InterProScan searches implemented in BLAST2GO PRO v4.1 (www.blast2go.de).

### PCR

Primers were obtained from Invitrogen (Life Technologies) and reactions were carried out using thermocyclers PeqStar (PEQLAB Biotechnology) and TProfessional Gradient (Biometra). Unless otherwise noted, reactions were carried out in a volume of 50 µl Dream Taq buffer supplemented with 0.2 mM of each dNTP, 0.2 mM of each primer, 1 µl cDNA and 0.25 µl DreamTaq Polymerase (Thermo Scientific) and were subjected to the following temperature program: 95 °C for 5 min, 35 cycles of 95 °C for 45 s, appropriate annealing temperature for 1 min, and 72 °C for 1 min, and a final incubation at 72 °C for 10 min. Primers are listed in Supplementary Table S2. Routine sequencing was done at Eurofins MWG Operon (Ebersberg, Germany).

### cDNA cloning

Sequence information of two transcripts annotated as thiosulfate-sulfurtransferase was retrieved from the *P*. *rapae* transcriptome (see Supplementary Fig. S1). To obtain full-length cDNAs of *PrTST1* and *PrTST2*, total RNA was isolated from about 500 µl of frozen and ground larval gut tissue as described^51^. 5 µg of RNA were used for cDNA synthesis using an Oligo(dT)_20_ primer and the 55Scriptase (Nippon Genetics) according to the manufacturer’s protocol. Gene-specific primer pairs P6/P7 and P8/P9 were used for PCR to confirm the two fragments identified in the database. For amplification of cDNA 3′-ends, primer anchor-(dT)_18_ was used for cDNA synthesis from RNA of gut tissue. This cDNA was used as a template in a 3′-RACE with a gene-specific primer (P10 or P12) and the anchor primer. To obtain the cDNA 5′ ends, the SMARTer RACE cDNA amplification kit (Clontech) was used. First-strand cDNA was synthesized using gene-specific primer P11 or P13 and the SMARTer IIa oligonucleotide according to the manufacturer’s instructions. This cDNA was used as a template in 5′-RACE with a gene-specific primer (P11 or P13) and the RACElong oligonucleotide. For *PrTST2* this was followed by a nested PCR with the RACEshort primer and the gene-specific primer P14. PCR products were cloned into pGEM-T Easy (Promega) according to the manufacturer’s instructions and sequenced. The complete coding sequences of *PrTST1* and *PrTST2* were confirmed by independent PCR amplification using primers P17/P18 and P15/16, respectively, and larval gut cDNA as a template (see Supplementary Fig. S1).

### Generation of expression constructs

Expression constructs were generated using a modified pET52b(+)vector (Novagen) as described previously^26^. The ORF was amplified from cDNA generated with larval gut RNA, Oligo(dT)_20_ primer and the 55Scriptase (Nippon Genetics) by PCR. Reaction mixtures contained 1 µl cDNA, primer pairs P17/P18 and P20/P21, respectively, and 0,5 µl *Pfu*Turbo Cx Hotstart Polymerase (Agilent) in a total volume of 25 µl. The PCR products were purified, cloned into the vector and sequenced. The expression construct for PrTST1 without the target peptide was produced in the same way using a PCR product generated with primers P18/P19 and cDNA as a template.

### Heterologous expression and purification of recombinant proteins

For heterologous expression of the recombinant enzymes, *E*. *coli* BL21 (DE3) pLysS (Invitrogen) were transformed with the expression constructs or the empty vector as a control. Expression, extraction and purification were done as described previously^26^. The elution fractions from Strep Tactin sepharose resin (IBA, Göttingen, Germany) were analyzed via SDS-PAGE and the fractions with the highest amounts of purified recombinant proteins (or the corresponding fractions from vector control samples) were pooled and used in enzyme assays. Protein concentrations were determined using the BCA Protein Assay Kit (Thermo Fisher Scientific) according to the manufacturer’s instructions.

### Rhodanese assay

Purified protein (125 µl, in 50 mM Tris-HCl, pH 8.5) was mixed with 250 µl 125 mM KCN and 250 µl 125 mM sodium thiosulfate (both in 100 mM Tris-HCl, pH 8.5) and incubated at 30 °C for 10–60 min^52,53^. The reaction was stopped by the addition of 125 µl formaldehyde and 625 µl ferric nitrate reagent^52^. After centrifugation for 5 min at 1,050 × g, the absorption of the supernatant was measured at 460 nm. The absorption of a standard dilution series was determined, using concentrations of 0–1,500 µM KSCN in 100 mM Tris-HCl, pH 8.5.

To verify the formation of thiocyanate, the enzymatic reaction was stopped by addition of 100 µl formaldehyde and centrifuged at 22,000 × g. The supernatant was analyzed using an HP1200 series HPLC instrument (Agilent Technologies, Waldbronn, Germany) equipped with a Hypercarb column (50 × 3 mm, 5 µM particle size, 250 Å pore size; Thermo Scientific, Darmstadt, Germany) and coupled to a 3,200 QTRAP mass spectrometer (ABSciex). The mobile phase was composed of solvent A (2 mM ammonium acetate in water) and solvent B (95% (v/v) acetonitrile/5% (v/v) 2 mM ammonium acetate in water) and used at a flow rate of 0.5 ml/min. The gradient was as follows: 5% (v/v) B (1 min), 5–100% (v/v) B (0.1 min), 100% (v/v) B (1.9 min), 100–5% (v/v) B (0.1 min), 5% (v/v) B (1.9 min). Mass spectra were recorded starting at 1 min. The mass spectrometer was operated in the negative mode with source voltage and declustering potential of −4.5 kV and −50 V, respectively. The source temperature was 500 °C. Nitrogen was used for collision-induced dissociation at the medium setting. The collision energy was −5 V. The thiocyanate ion (m/z 57.9) was monitored.

For determination of kinetic parameters, the protein amount was varied to ensure a linear increase of the product amount within the incubation time. The protein amounts used for the kinetic characterization was 0.4 µg and 1 µg for PrTST1 and PrTST2, respectively. The kinetics of thiosulfate were measured in the presence of 50 mM KCN with varying amounts of thiosulfate, the kinetics of cyanide in the presence of 50 mM thiosulfate with varying amounts of cyanide. The means of three technical replicates were used for nonlinear fitting to the Michaelis-Menten equation using OriginPro 8. Three independent experiments were conducted to obtain mean *K*_m_ and V_max_ values as given in Table 2.

### Quantitative real-time PCR (qPCR)

Total RNA was isolated from larval tissues pooled from three late-instar larvae and three butterflies, respectively. After DNase treatment and supplementation with RNase inhibitor, 1 µg of RNA was used for cDNA synthesis. qPCR was performed in a total volume of 10 µl (5 µl Bio-Rad iTaq Universal SYBR Green Supermix, 0.5 µl of each gene-specific primer (Supplementary Table S2) and 4 µl of 400-fold diluted cDNA) using a Bio-Rad CFX Connect Real-Time PCR Detection System with the following temperature program: 30 s of 95 °C, 35 cycles of 5 s 95 °C/30 s 60 °C. Three independent biological samples were analyzed, each with three technical replicates. Primer efficiencies were determined using a serial dilution of pooled cDNA of all samples. The efficiencies were as followed: 0.963 (*EF1*_*α*_), 0.837 (*GAPDH*), 0.901 (*PrTST1*), 0.985 (*PrTST2*). The CT values of *PrTST1* and *PrTST2* (Supplementary Table S3) were normalized to either *EF1α* or *GAPDH* as reference genes using the following equation^26^:

$${(1+E)}^{-{\rm{\Delta }}CT}={(1+{{\rm{E}}}_{{\rm{Ref}}})}^{{\rm{CTRef}}}/{(1+{{\rm{E}}}_{{\rm{GOI}}})}^{{\rm{CTGOI}}}$$

Water controls were included. The identity of PCR products was confirmed by melting curve analysis as well as cloning and sequencing.

### Phylogenetic analysis

Amino acid sequences of putative rhodaneses from *P*. *rapae* were used as query sequences for BLAST searches (using tBLASTn with default algorithm parameter settings) in Genbank (Nucleotide collection (nr)), Whole Genome Sequencing database (WGS), Lepbase and in-house transcriptome assemblies. Sequences with more than 35% amino acid sequence similarity to queries were collected for further analyses (Supplementary Table S1). All protein sequences were aligned in Geneious (vR11) using MUSCLE^54^ with default settings, inspected for regions of high-quality alignment and refined manually. During this step, candidates were also scrutinized for the presence of conserved rhodanese amino acid patterns. Phylogenetic trees were generated based on the protein sequence alignments in Geneious using a Maximum Likelihood method and Bayesian inference. The PhyML method was used selecting the LG model, estimated gamma distribution parameters and fixed proportion of invariant sites. For gene tree generation using Bayesian inference, the prior was set for the amino acid models to mix, thereby allowing jumps between fixed-rate amino acid models and gamma distributed rate variation selected. Markov Chain Monte Carlo runs were carried out for 2,500,000 generations after which log likelihood values showed that equilibrium had been reached after the first 10,000 generations in all cases, and those data were discarded from each run and considered as ‘burnin’. Two runs were carried out for the dataset showing agreement in topology and likelihood scores. The neighbor-joining and Bayesian tree topologies were in agreement, including their general subfamily relationships and node supports.

### Data availability

Sequences identified in this study are available at Genbank (accession numbers MH036751 (PrTST1) and MH036752 (PrTST2)).

## Electronic supplementary material

Supplementary Information

Dataset 1
