# Supplementary material for: Molecular identification and characterization of rhodaneses from the insect herbivore Pieris rapae
Source: Sci Rep. 2018 Jul 17;8:10819. doi: 10.1038/s41598-018-29148-5 (PMC6050342; doi:10.1038/s41598-018-29148-5)
Supplement: Supplementary file 1 — Supplementary Information [file 41598_2018_29148_MOESM1_ESM.pdf]

SUPPLEMENTARY INFORMATION

Molecular identification and characterization of rhodaneses from the insect herbivore *Pieris rapae*

Anna-Maria Steiner, Christine Busching, Heiko Vogel and Ute Wittstock

a tgaaattttattataaatagtagtcaattttacattttacgcatatttagtaggtttaattcct
*taa*tccttgacctatcaaaaccatcgttatttcaat**ATG**ATCGGTCGTATTTTATCTCGCCGCACATTG
GCGACATCTTATCAAGTTTTGAAAATATTATCAAATCAAAGTTTTATTTCATCAACCAGAAATGGTTCATT
ACAGTCGTTTAACTATCCAACTTTAAAAAATTCACTAGTTCGCACATATGCAGAACAAGTTAAAGTTGAAG
ATGTTACAGTTGACTTTGAGTATGTTAATAAAGCAATAAATAATCGCAATATCCTAATTATTGATGTCCGG
GAACCTGATGAAATTAAGGAACATGGAAAAATTCCGAATAGTATTAACATACCATTGGGCAATGTAACACC
CGTACTTAGTACTATGCCAGAAAATGAATTTGCTAAACAATATAATAAAGAGAAACCTTCAGATGACAGTG
AAATAATTTTTTATTGTATGATTGGCAAAAGATCGGGTATGGCTCAGCAGAATGCTATCAACCTTGGTT
ATAAAAATGCTAAGAATTATGTTGGAAGTTATACAGATTGGGCAAGTAAAACACAG**TGA**aattt
gacaataaatgttatatagtttggaagaattggtgattggtaatattgactttggttattgaat
ataattagaaaattctgttagacctgcttgtaaagtacatacttgaactgtatctatcataaat
aatgttgtaaatgaattatactatatttaatttaccatataatattaaatcaaattaaatgcat
aaatatatttttgtaaataaattaaatcttaacgccataactgctaaatcctatactgtatat
atttgtattaattaatgggttgaccagtcatcatagctcccaatgtacactttagacctgaaat
aaagtttatatcaagaaaaaaaaaaaaaaaaaa

b acgcggggagcaaataaaatattgaagtgttaaacaatagtacctatagttctagcaattttaCAGGTATA
CGAAGATATATTTACAAGC*TAA*TATTTAAGATTTTGTTGTTTCTTTTGTATCAGTTGTGATCATTTTAAGT
TA**ATG**GGTAATTCAAATAGCAATAGCACAATGGTCGACCCGGTGAGATTTGTGAGTTATGAAGACATGTTG
AAGGTCATTCACGAGCCCGAGAAAGTGGTAATTGATGTACGCAATCCAGATGAAATTGAATCAACT
GGCAAAATTCCTTCCAGTATAAATATACCTTTAAACACTGTATCCGACACTCTTGCGTCGATGC
CTGATGATGAGTTCCTGAAACAGTACCAACGACCAAAGCCGTCAGCATCTGACGAGTTAATTTT
CTACTGCAAATCCGGGAGACGTTCTCAAGAGGCCCTTGACAAAGCATTGAAACTGGGATTTTCAAACTCC
AAGTCATACTTAGGAAGTTGGGAGGACTGGTCAAGCAAGCAGAAG**TAA**ACAATCACTGGAGAAGACATCTT
CAATCGATGaagagatcaatcaccacttttatctttttacagttatgctataattttaaggactaaaataa
atatttgcgcaaaaaaaaaaaaaaaaaaaaaa

Figure S1. *PrTST1* and *PrTST2* cDNA sequences (5'--> 3'). The sequences of the RNAseq database entries together with the extensions obtained by 5'- and 3'-RACE (shaded) is shown for *PrTST1* (a) and *PrTST2* (b). The ORF is underlined, start and stop codon are printed in bold. The first upstream in-frame stop codon is printed in italics. Sequence confirmed in a single PCR is represented in upper case letters.

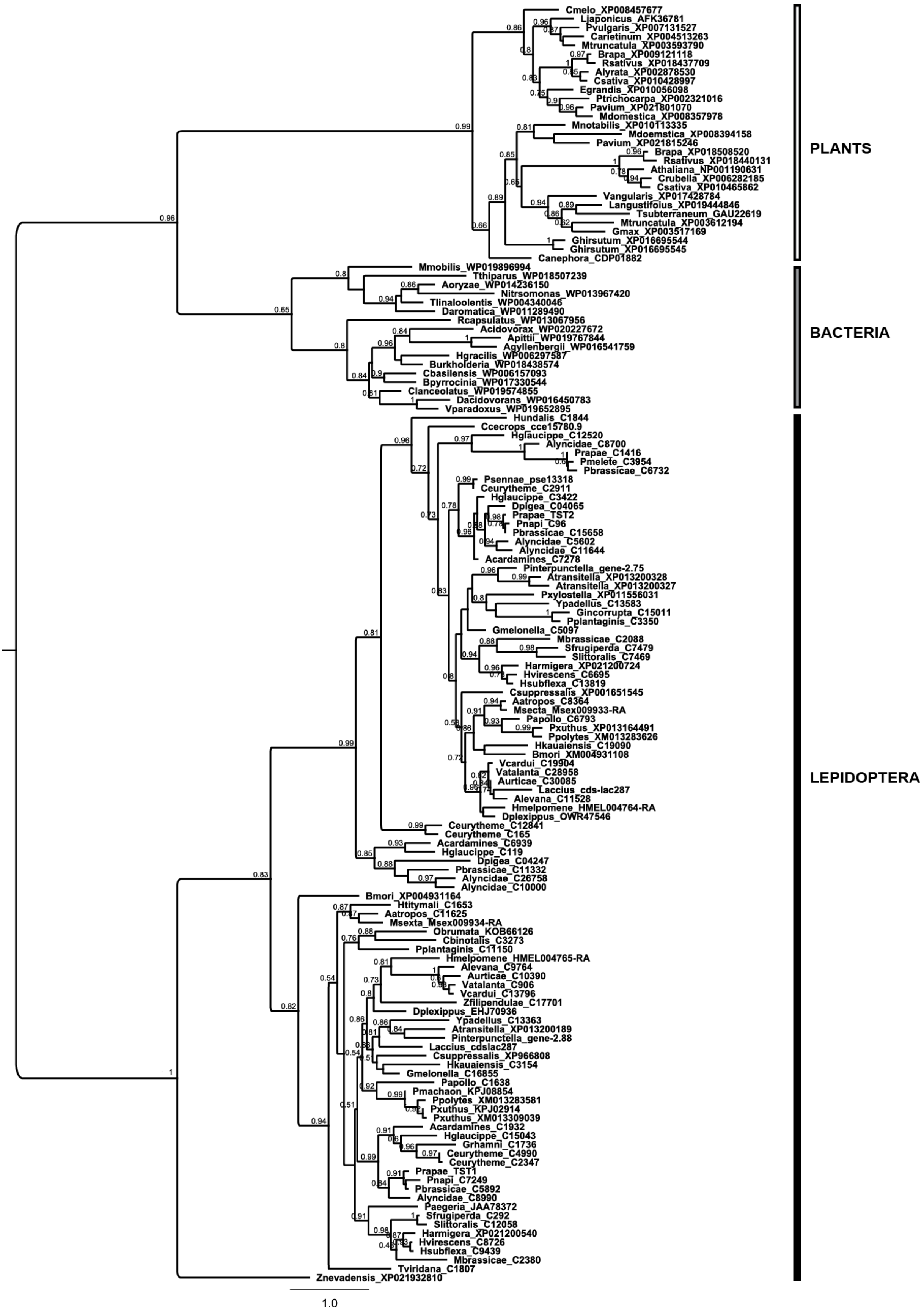

Figure S2. Phylogenetic relationships of rhodaneses and putative rhodaneses from Lepidoptera, Bacteria and plants. A maximum likelihood analysis was performed with 1,000 bootstrap replicates with bootstrap values indicated next to the branches. The tree is represented as a cladogram with branch lengths with the *Zootermopsis nevadensis*

(Isoptera) rhodanese sequence (GenBank Accession Number XP021932810) as outgroup for the Lepidopteran sequences. Details of the species names and sequences used for the analysis as well as accession numbers are provided in Supplementary Table S1 online.

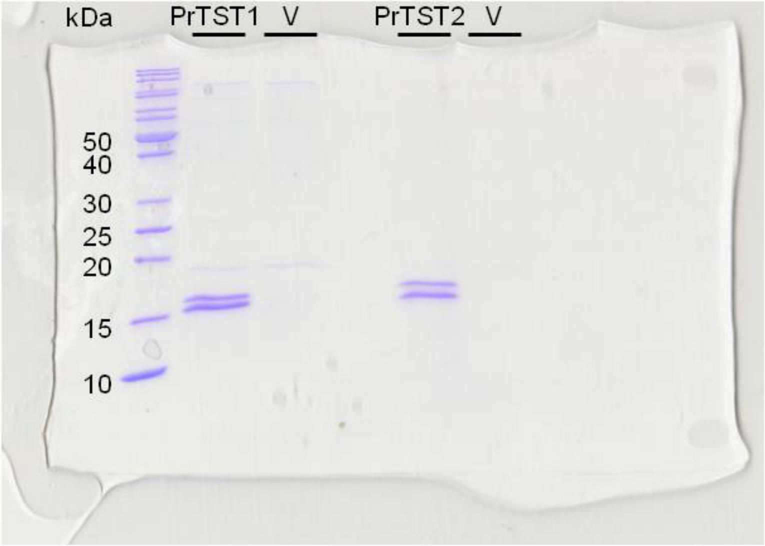

Figure S3. Image of full-length SDS-PAGE gel of purified PrTST1 and PrTST2. Proteins were expressed in E. coli in fusion with an N-terminal Strep-tag (PrTST1 without its target peptide). As a control, *E. coli* transformed with the expression vector without insert were treated the same. SDS-PAGE of 2 µg of purified recombinant enzymes compared with the same volumes of vector control (V).

Table S2. Oligonucleotides used for cloning, RACE, and qPCR.

Abbr.	Name	Sequence (5‘-3‘)
P1	anchor-oligo(dT)$_{18}$	GGCCACGCGTCGACTAGTACTTTTTTTTTTTTTTTTTT
P2	anchor	GGCCACGCGTCGACTAGTAC
P 3	SMARTer IIA	AAGCAGTGGTAACAACGCAGAGTACGCGGG
P 4	RACElong	CTAATACGACTCACTATAGGGCAAGCAGTGGTATCAACGCAGAGT
P 5	RACEshort	CTAATACGACTCACTATAGGGC
P 6	RhoA for1	ACCATCGTTATTTCAATATGATCG
P 7	RhoA rev1	ACCAAGGTTGATAGCATTCTGC
P 8	RhoB for1	GGTGAGATTTGTGAGTTATGAAGAC
P 9	RhoB rev1	CAGTTTCAATGCTTTGTCAAGG
P 10	Rho1for	AAAGATCGGGTATGGCTCAGC
P 11	Rho1rev2	ATGTGCGGCGAGATAAAATACG
P 12	Rho2for	ATCCGGGAGACGTTCTCAAGAG
P 13	Rho2 rev3	CGTCGATGCCTGATGATGAGTT
P 14	Rho2 rev1	CATTCACGAGCCCGAGAA
P 15	Rho2 full for	CAGGTATACGAAGATATATTTACAAGC
P 16	Rho2 full rev	CATCGATTGAAGATGTCTTCTCC
P 17	Rho1 for USER	GGCTTAAUATGATCGGTCGTATTTTATC
P 18	Rho1 rev USER	GGTTTAAUTCACTGTGTTTTACTTGCC
P 19	Rho1 oSP for USER	GGCTTAAUATGGCAGAACAAGTTAAAG
P 20	Rho2 USER for	GGCTTAAUATGGGTAATTCAAATAGCAATAGC
P 21	Rho2 USER rev	GGTTTAAUTTACTTCTGCTTGCTTGACC
P 22	Rho1 for1 qPCR	TTGGGCAATGTAACACCCGT
P 23	Rho1 rev1 qPCR	ATTCTGCTGAGCCATACCCG
P 24	Rho2 for1 qPCR	GAGACGTTCTCAAGAGGCCC
P 25	Rho2 rev1 qPCR	ACCAGTCCTCCCAACTTCCT
P 26	qPCR Ef1a for	CTGGCACGGAGACAACAT
P 27	qPCR Ef1a rev	GCAGGTGGCAAGATAGCA
P 28	qPCR GAPDH for	ACATTATTCCTGCTGCTACTGG
P 29	qPCR GAPDH rev	GACAGTCAAATCCACAACAGAGA

Table S3. qPCR raw data.

	Target	Sample	CT
Test of primer efficiency	Rho1	undiluted	22.55
	Rho1	undiluted	22.4
	Rho1	undiluted	22.48
	Rho2	undiluted	19.17
	Rho2	undiluted	19.03
	Rho2	undiluted	18.98
	Ef1α	undiluted	16.34
	Ef1α	undiluted	16.23
	Ef1α	undiluted	16.36
	GAPDH	undiluted	20.12
	GAPDH	undiluted	19.74
	GAPDH	undiluted	19.92
	Rho1	1to10	25.75
	Rho1	1to10	25.77
	Rho1	1to10	25.64
	Rho2	1to10	22.25
	Rho2	1to10	22.27
	Rho2	1to10	22.19
	Ef1α	1to10	19.6
	Ef1α	1to10	19.61
	Ef1α	1to10	19.83
	GAPDH	1to10	23.37
	GAPDH	1to10	23.45
	GAPDH	1to10	23.46
	Rho1	1to100	29.38
	Rho1	1to100	29.11
	Rho1	1to100	29.06
	Rho2	1to100	25.5
	Rho2	1to100	25.72
	Rho2	1to100	25.71
	Ef1α	1to100	23.12
	Ef1α	1to100	23.06
	Ef1α	1to100	23.1
	GAPDH	1to100	27.16
	GAPDH	1to100	27.24
	GAPDH	1to100	27.42
	Rho1	1to1.000	34.04
	Rho1	1to1.000	33.29
	Rho1	1to1.000	32.79
	Rho2	1to1.000	29.11
	Rho2	1to1.000	29.4
	Rho2	1to1.000	29.31
	Ef1α	1to1.000	26.41

	Ef1α	1to1.000	26.61
	Ef1α	1to1.000	26.47
	GAPDH	1to1.000	31.22
	GAPDH	1to1.000	31.26
	GAPDH	1to1.000	31.17
	Rho1	1to10.000	N/A
	Rho2	1to10.000	31.96
	Rho2	1to10.000	32.49
	Ef1α	1to10.000	29.79
	Ef1α	1to10.000	30.19
	GAPDH	1to10.000	34.89
	GAPDH	1to10.000	35.12
	GAPDH	1to10.000	35.11
	Rho1	NTC	N/A
	Rho1	NTC	N/A
	Rho1	NTC	N/A
	Rho2	NTC	N/A
	Rho2	NTC	N/A
	Rho2	NTC	N/A
	Ef1α	NTC	N/A
	Ef1α	NTC	N/A
	Ef1α	NTC	N/A
	GAPDH	NTC	N/A
	GAPDH	NTC	N/A
	GAPDH	NTC	N/A
Expression analysis	Rho1	head1	30.02
	Rho1	head1	30.15
	Rho1	head1	30.1
	Rho2	head1	28.12
	Rho2	head1	28.04
	Rho2	head1	28.24
	EF1α	head1	23.38
	EF1α	head1	23.45
	EF1α	head1	23.45
	Rho1	gut1	29.52
	Rho1	gut1	29.26
	Rho1	gut1	29.53
	Rho2	gut1	25.07
	Rho2	gut1	24.93
	Rho2	gut1	24.98
	EF1α	gut1	24.59
	EF1α	gut1	24.54
	EF1α	gut1	24.49
	Rho1	integument1	30.85
	Rho1	integument1	31.55

	Rho1	integument1	31.21
	Rho2	integument1	30.23
	Rho2	integument1	30.21
	Rho2	integument1	30.61
	EF1α	integument1	24.79
	EF1α	integument1	24.7
	EF1α	integument1	24.82
	Rho1	head2	30.21
	Rho1	head2	29.7
	Rho1	head2	30.17
	Rho2	head2	27.65
	Rho2	head2	27.88
	Rho2	head2	27.66
	EF1α	head2	23.09
	EF1α	head2	23.12
	EF1α	head2	23.08
	Rho1	gut2	28.69
	Rho1	gut2	29.07
	Rho1	gut2	28.92
	Rho2	gut2	24.36
	Rho2	gut2	24.36
	Rho2	gut2	24.23
	EF1α	gut2	24.24
	EF1α	gut2	24.34
	EF1α	gut2	24.16
	Rho1	integument2	30.54
	Rho1	integument2	30.53
	Rho1	integument2	30.67
	Rho2	integument2	30.56
	Rho2	integument2	30.33
	Rho2	integument2	30.26
	EF1α	integument2	23.7
	EF1α	integument2	23.67
	EF1α	integument2	23.79
	Rho1	NTC	N/A
	Rho1	NTC	36.14
	Rho1	NTC	N/A
	Rho2	NTC	N/A
	Rho2	NTC	N/A
	Rho2	NTC	N/A
	EF1α	NTC	N/A
	EF1α	NTC	N/A
	EF1α	NTC	N/A
	Rho1	head3	30.33
	Rho1	head3	30.19

	Rho1	head3	30.22
	Rho2	head3	28.08
	Rho2	head3	27.95
	Rho2	head3	28.11
	EF1α	head3	23.75
	EF1α	head3	23.8
	EF1α	head3	23.78
	Rho1	gut3	29.23
	Rho1	gut3	29.01
	Rho1	gut3	29.11
	Rho2	gut3	24.21
	Rho2	gut3	24.23
	Rho2	gut3	24.18
	EF1α	gut3	24.32
	EF1α	gut3	24.28
	EF1α	gut3	24.27
	Rho1	integument3	30.14
	Rho1	integument3	29.94
	Rho1	integument3	29.87
	Rho2	integument3	30.11
	Rho2	integument3	30.28
	Rho2	integument3	30.27
	EF1α	integument3	23.45
	EF1α	integument3	23.53
	EF1α	integument3	23.56
	Rho1	butterfly1	30.91
	Rho1	butterfly1	31.15
	Rho1	butterfly1	30.82
	Rho2	butterfly1	32.38
	Rho2	butterfly1	32.61
	Rho2	butterfly1	32.08
	EF1α	butterfly1	24.17
	EF1α	butterfly1	24.18
	EF1α	butterfly1	24.17
	Rho1	butterfly2	30.14
	Rho1	butterfly2	30.05
	Rho1	butterfly2	29.74
	Rho2	butterfly2	32.99
	Rho2	butterfly2	34.69
	Rho2	butterfly2	33.03
	EF1α	butterfly2	23.81
	EF1α	butterfly2	23.92
	EF1α	butterfly2	23.83
	Rho1	butterfly3	29.35
	Rho1	butterfly3	29.74

	Rho1	butterfly3	29.18
	Rho2	butterfly3	33.37
	Rho2	butterfly3	33.16
	Rho2	butterfly3	32.93
	EF1α	butterfly3	22.42
	EF1α	butterfly3	22.8
	EF1α	butterfly3	22.58
	Rho1	NTC	N/A
	Rho1	NTC	N/A
	Rho1	NTC	35.13
	Rho2	NTC	N/A
	Rho2	NTC	N/A
	Rho2	NTC	N/A
	EF1α	NTC	N/A
	EF1α	NTC	N/A
	EF1α	NTC	N/A
	GAPDH	head1	27.28
	GAPDH	head1	27.75
	GAPDH	head1	27.28
	GAPDH	head3	28.09
	GAPDH	head3	28.04
	GAPDH	head3	28.05
	GAPDH	gut1	29.75
	GAPDH	gut1	29.34
	GAPDH	gut1	29.35
	GAPDH	gut3	29.54
	GAPDH	gut3	29.61
	GAPDH	gut3	29.23
	GAPDH	integument1	28.3
	GAPDH	integument1	28.36
	GAPDH	integument1	28.14
	GAPDH	integument1	27.94
	GAPDH	integument1	27.64
	GAPDH	integument1	27.79
	GAPDH	head2	27.71
	GAPDH	head2	27.39
	GAPDH	head2	27.97
	GAPDH	butterfly1	28.42
	GAPDH	butterfly1	28.16
	GAPDH	butterfly1	28.36
	GAPDH	gut2	29.55
	GAPDH	gut2	29.65
	GAPDH	gut2	29.38
	GAPDH	butterfly2	28.72
	GAPDH	butterfly2	28.62

	GAPDH	butterfly2	28.51
	GAPDH	integument2	28.48
	GAPDH	integument2	28.11
	GAPDH	integument2	28.14
	GAPDH	butterfly3	27.87
	GAPDH	butterfly3	27.73
	GAPDH	butterfly3	27.68
	GAPDH	NTC	N/A
	GAPDH	NTC	N/A
	GAPDH	NTC	N/A
